# Supplementary material for: Is the Magnesium Content in Food Supplements Consistent with the Manufacturers’ Declarations?
Source: Nutrients. 2021 Sep 28;13(10):3416. doi: 10.3390/nu13103416 (PMC8538386; doi:10.3390/nu13103416)
Supplement: Supplementary file 1 [file nutrients-13-03416-s001.zip › nutrients-1398396-supplementary.pdf]

nutrients

MDPI

Supplementary Materials

Is The Magnesium Content In Dietary Supplements Consistent With The Manufacturers' Declarations?

Anna Puścion-Jakubik [#,*], Natalia Bartosiewicz [#] and Katarzyna Socha

Department of Bromatology, Faculty of Pharmacy with the Division of Laboratory Medicine, Medical University of Białystok, Mickiewicza 2D Street, 15-222 Białystok, Poland; natalia_bartosiewicz@wp.pl (N.B.); katarzyna.socha@umb.edu.pl (K.S.)
* Correspondence: anna.puscion-jakubik@umb.edu.pl; Tel.: +48-8574-854-69
# These authors contributed equally to this work.

Table S1. Characteristics of the studied dietary supplements.

Number	Declared content of Mg in 1 portion [mg]	Form	Price	Declared content of Mg in 1 portion [mg]	Amount of minerals	Chemical form
1.	17	Tablets	<10	Less than 100 mg	Multicomponent preparations	Magnesium carbonate
2.	28.5	Jelly beans	from 10 to 20	Less than 100 mg	Multicomponent preparations	Magnesium carbonate
3.	30	Coated tablets	from 10 to 20	Less than 100 mg	Multicomponent preparations	Magnesium citrate
4.	30	Capsules	from 10 to 20	Less than 100 mg	Multicomponent preparations	Magnesium citrate
5.	30	Tablets	from 10 to 20	Less than 100 mg	Multicomponent preparations	Magnesium carbonate
6.	30	Tablets	from 10 to 20	Less than 100 mg	Only magnesium (or vitamin B6)	Several chemical forms
7.	30	Tablets	from 10 to 20	Less than 100 mg	Only magnesium (or vitamin B6)	Magnesium lactate
8.	35	Tablets	from 10 to 20	Less than 100 mg	Only magnesium (or vitamin B6)	Magnesium citrate

9.	37.5	Tablets	<10	Less than 100 mg	Only magnesium (or vitamin B6)	Magnesium citrate
10.	45	Effervescent tablets	from 10 to 20	Less than 100 mg	Only magnesium (or vitamin B6)	Magnesium carbonate
11.	45	Effervescent tablets	from 10 to 20	Less than 100 mg	Multicomponent preparations	Magnesium carbonate
12.	50	Coated tablets	from 10 to 20	Less than 100 mg	Multicomponent preparations	Magnesium citrate
13.	50	Coated tablets	from 10 to 20	Less than 100 mg	Multicomponent preparations	Magnesium oxide
14.	50	Coated tablets	<10	Less than 100 mg	Only magnesium (or vitamin B6)	Magnesium lactate
15.	50	Coated tablets	from 10 to 20	Less than 100 mg	Multicomponent preparations	Magnesium carbonate
16.	50	Capsules	>20	Less than 100 mg	Multicomponent preparations	Several chemical forms
17.	50	Tablets	<10	Less than 100 mg	Only magnesium (or vitamin B6)	Magnesium citrate
18.	50	Coated tablets	<10	Less than 100 mg	Multicomponent preparations	Magnesium carbonate
19.	51	Tablets	<10	Less than 100 mg	Multicomponent preparations	Magnesium citrate
20.	51	Tablets	<10	Less than 100 mg	Only magnesium (or vitamin B6)	Magnesium lactate
21.	56.25	Tablets	from 10 to 20	Less than 100 mg	Multicomponent preparations	Magnesium oxide
22.	56.25	Tablets	from 10 to 20	Less than 100 mg	Only magnesium (or vitamin B6)	Magnesium lactate
23.	56.25	Powders	from 10 to 20	Less than 100 mg	Multicomponent preparations	Magnesium carbonate
24.	56.25	Tablets	<10	Less than 100 mg	Only magnesium (or vitamin B6)	Magnesium carbonate
25.	56.25	Coated tablets	<10	Less than 100 mg	Only magnesium (or vitamin B6)	Magnesium lactate
26.	56.25	Dragees	>20	Less than 100 mg	Multicomponent preparations	Magnesium carbonate
27.	56.5	Coated tablets	<10	Less than 100 mg	Multicomponent preparations	Magnesium carbonate
28.	58	Capsules	from 10 to 20	Less than 100 mg	Only magnesium (or vitamin B6)	Magnesium lactate
29.	60	Coated tablets	<10	Less than 100 mg	Multicomponent preparations	Magnesium carbonate
30.	60	Effervescent tablets	from 10 to 20	Less than 100 mg	Multicomponent preparations	Magnesium carbonate
31.	60	Tablets	<10	Less than 100 mg	Only magnesium (or vitamin B6)	Several chemical forms

32.	60	Powders	from 10 to 20	Less than 100 mg	Multicomponent preparations	Magnesium carbonate
33.	60	Effervescent tablets	from 10 to 20	Less than 100 mg	Multicomponent preparations	Magnesium lactate
34.	60	Effervescent tablets	from 10 to 20	Less than 100 mg	Multicomponent preparations	Magnesium lactate
35.	60	Tablets	<10	Less than 100 mg	Multicomponent preparations	Magnesium hydroxide
36.	60	Tablets	<10	Less than 100 mg	Only magnesium (or vitamin B6)	Magnesium oxide
37.	60	Tablets	from 10 to 20	Less than 100 mg	Only magnesium (or vitamin B6)	Magnesium carbonate
38.	60	Effervescent tablets	from 10 to 20	Less than 100 mg	Only magnesium (or vitamin B6)	Magnesium lactate
39.	62.5	Liquids	>20	Less than 100 mg	Only magnesium (or vitamin B6)	Several chemical forms
40.	70	Tablets	from 10 to 20	Less than 100 mg	Only magnesium (or vitamin B6)	Magnesium carbonate
41.	72	Tablets	<10	Less than 100 mg	Multicomponent preparations	Several chemical forms
42.	72	Tablets	>20	Less than 100 mg	Only magnesium (or vitamin B6)	Magnesium glycer-ophosphate
43.	75	Liquids	<10	Less than 100 mg	Only magnesium (or vitamin B6)	Magnesium citrate
44.	75	Tablets	from 10 to 20	Less than 100 mg	Only magnesium (or vitamin B6)	Magnesium citrate
45.	90	Liquids	from 10 to 20	Less than 100 mg	Only magnesium (or vitamin B6)	Several chemical forms
46.	93.75	Capsules	from 10 to 20	Less than 100 mg	Only magnesium (or vitamin B6)	Magnesium citrate
47.	93.75	Effervescent tablets	<10	Less than 100 mg	Multicomponent preparations	Magnesium carbonate
48.	93.75	Capsules	>20	Less than 100 mg	Multicomponent preparations	Several chemical forms
49.	94	Capsules	from 10 to 20	Less than 100 mg	Only magnesium (or vitamin B6)	Several chemical forms
50.	100	Capsules	from 10 to 20	100 - 200 mg	Only magnesium (or vitamin B6)	Magnesium bisglycinate
51.	100	Tablets	>20	100 - 200 mg	Multicomponent preparations	Magnesium carbonate
52.	100	Tablets	>20	100 - 200 mg	Only magnesium (or vitamin B6)	Magnesium citrate

53.	100	Coated tablets	<10	100 - 200 mg	Only magnesium (or vitamin B6)	Magnesium citrate
54.	100	Capsules	from 10 to 20	100 - 200 mg	Only magnesium (or vitamin B6)	Magnesium bisglycinate
55.	100	Effervescent tablets	from 10 to 20	100 - 200 mg	Only magnesium (or vitamin B6)	Magnesium bisglycinate
56.	100	Capsules	>20	100 - 200 mg	Only magnesium (or vitamin B6)	Magnesium bisglycinate
57.	100	Tablets	>20	100 - 200 mg	Multicomponent preparations	Magnesium oxide
58.	100	Tablets	<10	100 - 200 mg	Multicomponent preparations	Magnesium carbonate
59.	100	Effervescent tablets	<10	100 - 200 mg	Only magnesium (or vitamin B6)	Magnesium carbonate
60.	100	Tablets	<10	100 - 200 mg	Only magnesium (or vitamin B6)	Magnesium citrate
61.	100	Tablets	from 10 to 20	100 - 200 mg	Only magnesium (or vitamin B6)	Magnesium carbonate
62.	101.25	Powders	<10	100 - 200 mg	Only magnesium (or vitamin B6)	Magnesium carbonate
63.	102	Tablets	from 10 to 20	100 - 200 mg	Only magnesium (or vitamin B6)	Magnesium citrate
64.	102	Capsules	<10	100 - 200 mg	Only magnesium (or vitamin B6)	Magnesium citrate
65.	102	Tablets	from 10 to 20	100 - 200 mg	Only magnesium (or vitamin B6)	Magnesium citrate
66.	102	Tablets	from 10 to 20	100 - 200 mg	Multicomponent preparations	Magnesium citrate
67.	110	Coated tablets	from 10 to 20	100 - 200 mg	Only magnesium (or vitamin B6)	Magnesium citrate
68.	112.5	Dragees	>20	100 - 200 mg	Multicomponent preparations	Several chemical forms
69.	120	Tablets	from 10 to 20	100 - 200 mg	Only magnesium (or vitamin B6)	Magnesium carbonate
70.	120	Effervescent tablets	from 10 to 20	100 - 200 mg	Only magnesium (or vitamin B6)	Magnesium carbonate
71.	125	Tablets	<10	100 - 200 mg	Only magnesium (or vitamin B6)	Magnesium carbonate
72.	125	Effervescent tablets	<10	100 - 200 mg	Multicomponent preparations	Magnesium carbonate
73.	125	Tablets	from 10 to 20	100 - 200 mg	Only magnesium (or vitamin B6)	Magnesium citrate
74.	125	Tablets	from 10 to 20	100 - 200 mg	Only magnesium (or vitamin B6)	Several chemical forms
75.	130	Tablets	<10	100 - 200 mg	Only magnesium (or vitamin B6)	Magnesium citrate

76.	135	Powders	from 10 to 20	100 - 200 mg	Only magnesium (or vitamin B6)	Magnesium citrate
77.	150	Powders	>20	100 - 200 mg	Only magnesium (or vitamin B6)	Magnesium citrate
78.	150	Effervescent tablets	from 10 to 20	100 - 200 mg	Only magnesium (or vitamin B6)	Magnesium citrate
79.	150	Effervescent tablets	from 10 to 20	100 - 200 mg	Multicomponent preparations	Magnesium carbonate
80.	150	Tablets	from 10 to 20	100 - 200 mg	Only magnesium (or vitamin B6)	Magnesium citrate
81.	150	Granulates	from 10 to 20	100 - 200 mg	Only magnesium (or vitamin B6)	Magnesium citrate
82.	150	Tablets	from 10 to 20	100 - 200 mg	Only magnesium (or vitamin B6)	Magnesium lactate
83.	187.5	Effervescent tablets	from 10 to 20	100 - 200 mg	Multicomponent preparations	Magnesium carbonate
84.	187.5	Effervescent tablets	<10	100 - 200 mg	Only magnesium (or vitamin B6)	Magnesium carbonate
85.	187.5	Powders	from 10 to 20	100 - 200 mg	Multicomponent preparations	Magnesium citrate
86.	187.5	Tablets	from 10 to 20	100 - 200 mg	Only magnesium (or vitamin B6)	Several chemical forms
87.	200	Powders	from 10 to 20	100 - 200 mg	Multicomponent preparations	Magnesium citrate
88.	200	Effervescent tablets	<10	100 - 200 mg	Only magnesium (or vitamin B6)	Magnesium carbonate
89.	200	Effervescent tablets	from 10 to 20	100 - 200 mg	Only magnesium (or vitamin B6)	Magnesium lactate
90.	200	Tablets	>20	100 - 200 mg	Only magnesium (or vitamin B6)	Several chemical forms
91.	200	Powders	from 10 to 20	100 - 200 mg	Only magnesium (or vitamin B6)	Several chemical forms
92.	200	Tablets	from 10 to 20	100 - 200 mg	Only magnesium (or vitamin B6)	Magnesium carbonate
93.	225	Tablets	>20	100 - 200 mg	Multicomponent preparations	Magnesium hydroxide
94.	225	Tablets	>20	100 - 200 mg	Multicomponent preparations	Magnesium citrate
95.	300	Powders	>20	Above 200 mg	Multicomponent preparations	Magnesium citrate
96.	300	Effervescent tablets	<10	Above 200 mg	Only magnesium (or vitamin B6)	Several chemical forms
97.	300	Liquids	<10	Above 200 mg	Multicomponent preparations	Several chemical forms

98.	300	Liquids	<10	Above 200 mg	Multicomponent preparations	Magnesium bisglycinate
99.	362	Powders	>20	Above 200 mg	Only magnesium (or vitamin B6)	Magnesium citrate
100.	375	Effervescent tablets	<10	Above 200 mg	Only magnesium (or vitamin B6)	Magnesium carbonate
101.	375	Effervescent tablets	<10	Above 200 mg	Only magnesium (or vitamin B6)	Several chemical forms
102.	375	Powders	from 10 to 20	Above 200 mg	Only magnesium (or vitamin B6)	Magnesium citrate
103.	375	Liquids	<10	Above 200 mg	Only magnesium (or vitamin B6)	Magnesium bisglycinate
104.	375	Liquids	<10	Above 200 mg	Only magnesium (or vitamin B6)	Magnesium citrate
105.	375	Capsules	<10	Above 200 mg	Only magnesium (or vitamin B6)	Magnesium oxide
106.	375	Capsules	<10	Above 200 mg	Only magnesium (or vitamin B6)	Magnesium oxide
107.	375	Powders	>20	Above 200 mg	Only magnesium (or vitamin B6)	Magnesium citrate
108.	375	Tablets	from 10 to 20	Above 200 mg	Only magnesium (or vitamin B6)	Magnesium citrate
109.	375	Effervescent tablets	<10	Above 200 mg	Only magnesium (or vitamin B6)	Magnesium carbonate
110.	375	Effervescent tablets	<10	Above 200 mg	Only magnesium (or vitamin B6)	Several chemical forms
111.	375	Effervescent tablets	from 10 to 20	Above 200 mg	Only magnesium (or vitamin B6)	Several chemical forms
112.	375	Tablets	<10	Above 200 mg	Only magnesium (or vitamin B6)	Magnesium oxide
113.	375	Effervescent tablets	from 10 to 20	Above 200 mg	Only magnesium (or vitamin B6)	Magnesium carbonate
114.	400	Capsules	>20	Above 200 mg	Only magnesium (or vitamin B6)	Several chemical forms
115.	400	Tablets	from 10 to 20	Above 200 mg	Multicomponent preparations	Magnesium oxide
116.	400	Tablets	<10	Above 200 mg	Only magnesium (or vitamin B6)	Magnesium citrate
